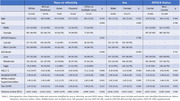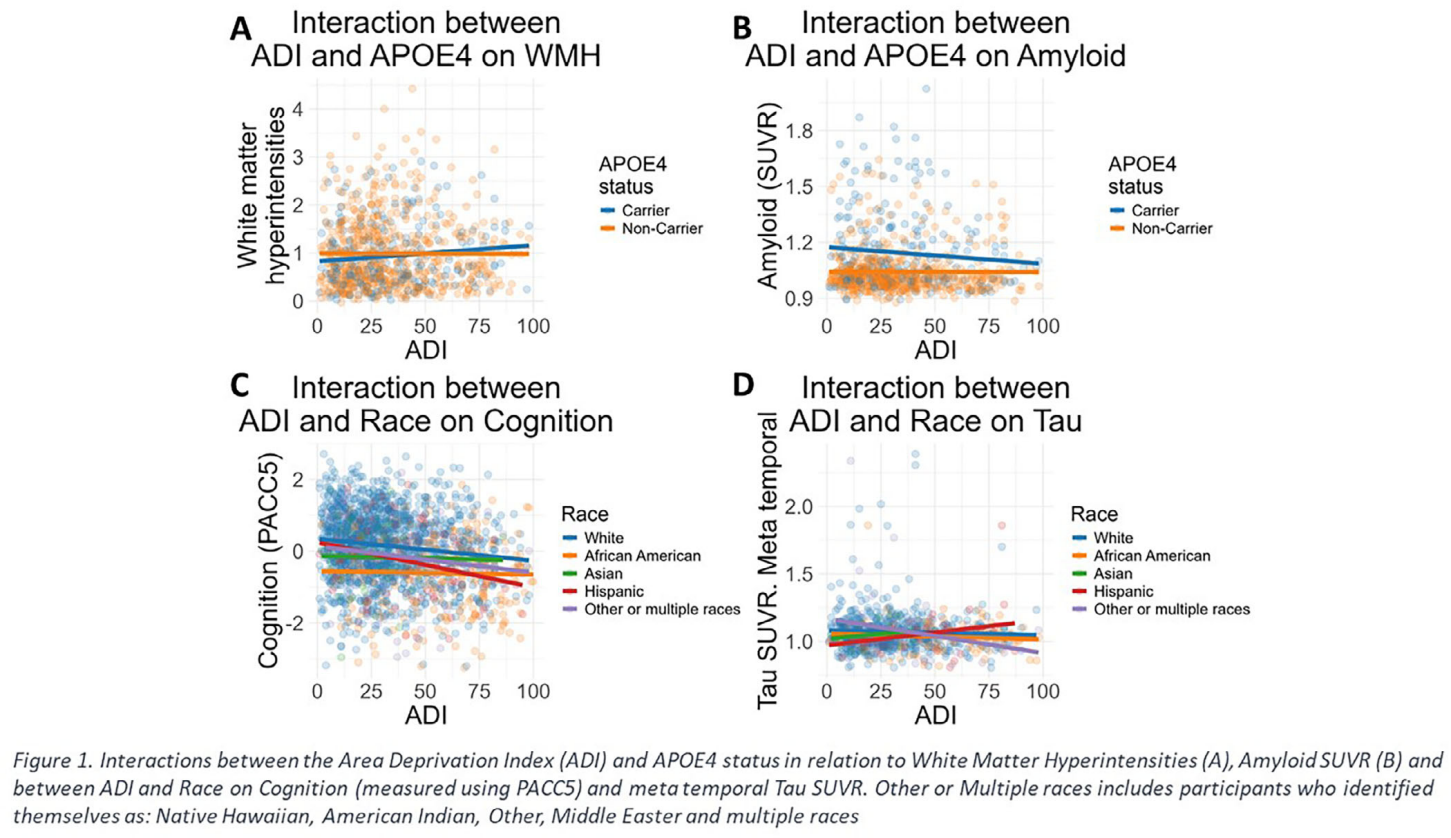# Association of neighborhood deprivation with Alzheimer's Disease pathologies, gray matter volume, white matter hyperintensities and cognition by sex, race or ethnicity and APOE4 status in Non‐demented Older Adults

**DOI:** 10.1002/alz70860_105535

**Published:** 2025-12-23

**Authors:** Pablo Aguilar, Thomas Monroe Holland, Sam N. Lockhart, Joseph C. Masdeu, Lycia Tramujas Vasconcellos Neumann, Heather M Snyder, Laura D Baker, Susan M. Landau, Eider M Arenaza‐Urquijo

**Affiliations:** ^1^ Global Health Institute Barcelona (ISGlobal), Barcelona, Spain; ^2^ University of Pompeu Fabra (UPF), Barcelona, Spain; ^3^ Sant Pau Memory Unit, Department of Neurology, Hospital de la Santa Creu i Sant Pau, Institut d'Investigació Biomèdica Sant Pau (IIB SANT PAU), Facultad de Medicina ‐ Universitat Autònoma de Barcelona, Barcelona, Spain; ^4^ Rush University Medical Center, Chicago, IL, USA; ^5^ Wake Forest University School of Medicine, Winston‐Salem, NC, USA; ^6^ Houston Methodist Research Institute, Houston, TX, USA; ^7^ Alzheimer's Association, Chicago, IL, USA; ^8^ Neuroscience Department, University of California, Berkeley, Berkeley, CA, USA; ^9^ Mayo Clinic, Rochester, MN, USA; ^10^ Centro de Investigación Biomédica en Red de Fragilidad y Envejecimiento Saludable (CIBERFES), Madrid, Spain; ^11^ ISGlobal ‐ Barcelona Institute for Global Health, Barcelona, Catalunya/Barcelona, Spain

## Abstract

**Background:**

Neighborhood socio‐economic conditions may increase Alzheimer's disease (AD) risk through amyloid, tau, atrophy or vascular pathways, potentially varying by genetic risk, race or ethnicity and sex. We examined associations between Area Deprivation Index (ADI) and cognition, AD pathology, gray matter (GM) and white matter hyperintensities (WMH) volume in cognitively unimpaired older adults by race or ethnicity, sex and APOE4 status.

**Method:**

We included 1896 non‐demented older adults from US POINTER study with available cognitive evaluations, ADI level – a census–based neighborhood disadvantage metric (categorized as low, moderate and high) and a subsample with neuroimaging data (*N* = 806). Linear/logistic regressions were used to assess interactions of ADI with sex, race or ethnicity, and APOE4 on (1) the Preclinical Alzheimer's Cognitive Composite, (PACC‐5), (2) MRI‐derived GM volume meta‐ROI (entorhinal, fusiform, parahippocampal, mid‐temporal, inferior temporal), (3) WMH and (4) AD pathology (amyloid and tau PET), adjusted by age, sex and intracranial volume when appropriate.

**Result:**

African American participants (*p* <0.001) and females (*p* <0.001) were more likely to live in deprived areas. African American participants had significantly lower PACC‐5 (*p* <0.001) and GM volume (*p* <0.001), “Other or multiple races” participants showed significantly higher amyloid burden (*p* = 0.001), and White participants higher WMH (*p* = 0.013). Females showed higher PACC‐5 scores (*p* <0.001), while APOE4 carriers showed lower PACC‐5 (*p* = 0.042) and higher amyloid burden (*p* <0.001) (Table 1). Race and ADI interacted on PACC‐5 (*p* = 0.043) and showed a borderline interaction on Tau (*p* = 0.079), with higher ADI linked to lower PACC‐5 in White, Hispanic, and “Other or Multiple Races” and higher Tau in Hispanic. Further, there was a borderline ADI*APOE4 status interaction on WMH (*p* = 0.059) and a significant ADI*APOE4 interaction on amyloid (*p* = 0.001), where APOE4 carriers in deprived areas showed higher WMH and lower amyloid burden (Figure 1). No significant ADI*sex interactions were observed.

**Conclusion:**

Neighborhood disadvantage was more commonly observed in African American participants, but interaction with cognition was primarily seen in other racial/ethnic groups. APOE4 carriers showed increased amyloid burden, but those from more disadvantaged neighborhoods exhibited a higher vascular component alongside lower amyloid‐driven pathology. These results may indicate varying effects of social disadvantages across groups or sample bias.